# Optimization of a New Design of Molten Salt-to-CO_2_ Heat Exchanger Using Exergy Destruction Minimization

**DOI:** 10.3390/e22080883

**Published:** 2020-08-12

**Authors:** María José Montes, José Ignacio Linares, Rubén Barbero, Beatriz Yolanda Moratilla

**Affiliations:** 1 E.T.S. Ingenieros Industriales-UNED, C/Juan del Rosal 12, 28040 Madrid, Spain; rbarbero@ind.uned.es; 2 Rafael Mariño Chair in New Energy Technologies–COMILLAS-ICAI, C/Alberto Aguilera 25, 28015 Madrid, Spain; linares@icai.comillas.edu (J.I.L.); ymoratilla@icai.comillas.edu (B.Y.M.)

**Keywords:** Solar Thermal Power Plants, supercritical CO2 cycles, MS-to-CO_2_ heat exchanger, thermo-economic optimization, exergy destruction minimization

## Abstract

One of the ways to make cost-competitive electricity, from concentrated solar thermal energy, is increasing the thermoelectric conversion efficiency. To achieve this objective, the most promising scheme is a molten salt central receiver, coupled to a supercritical carbon dioxide cycle. A key element to be developed in this scheme is the molten salt-to-CO_2_ heat exchanger. This paper presents a heat exchanger design that avoids the molten salt plugging and the mechanical stress due to the high pressure of the CO_2_, while improving the heat transfer of the supercritical phase, due to its compactness with a high heat transfer area. This design is based on a honeycomb-like configuration, in which a thermal unit consists of a circular channel for the molten salt surrounded by six smaller trapezoidal ducts for the CO_2_. Further, an optimization based on the exergy destruction minimization has been accomplished, obtained the best working conditions of this heat exchanger: a temperature approach of 50 °C between both streams and a CO_2_ pressure drop of 2.7 bar.

## 1. Introduction

The main advantages of the energy supplied through Solar Thermal Power Plants (STPPs) are its capacity, reliability and stability to the grid, which in turn allows the renewable electricity percentage to be also higher. However, when compared to the costs of solar photovoltaic electricity, the reduction in cost must still be very large for solar thermal electricity, to be competitive. One of the ways to achieve this is by increasing global conversion efficiency by coupling the solar field to a supercritical cycle. In this scheme, a reliable design of the heat exchanger between the solar field and the Brayton cycle is essential for the technical viability of these STPPs.

Within the SunShot program [1], the U.S. Department of Energy (DOE) has identified three potential schemes for the next generation of STPPs, based on the Heat Transfer Fluid (HTF) in the receiver: molten salts, falling particles or gas phase. In all the schemes, the solar field is coupled to a supercritical carbon dioxide (sCO_2_) cycle, achieving high thermo-electric conversion efficiency.

**Figure 1:**
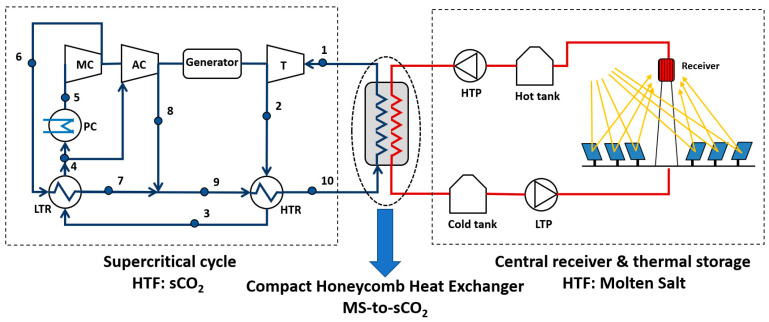
Scheme of the complete STPP (Solar Thermal Power Plants) with the CHHE (Compact Honeycomb Heat Exchanger) between the solar field and the sCO_2_ cycle.

The scheme based on a molten salt central receiver coupled to a sCO_2_ Brayton cycle is the most conventional one, as the molten salt systems are a state-of-art technology. Besides that, the Thermal Energy Storage (TES) associated, provides this scheme of a high capacity factor and a dispatchable electricity production [2]. This scheme is showed in Figure 1.

Nevertheless, several challenges arise in this technology, like an efficient central receiver working at a temperature higher than 700 °C; a supercritical cycle that maximizes performance and minimizes cost, taking into account the peculiarities of the solar field to which it is coupled; and, between these two subsystems, a key element is the heat exchanger (HX) to transfer energy from the molten salt in the solar field to the CO_2_ in the Brayton cycle, the Source Heat Exchanger (SHX). This paper deals in depth with this last equipment, proposing a design that can overcome some of the technological difficulties of these type of HXs: the mechanical stress due to the high pressure of the supercritical phase; the need of improving the heat transfer of the supercritical fluid; and overall, the molten salt plugging in the microchannels of a Compact Heat Exchanger (CHX), as it will be explained below.

Supercritical CO_2_ Brayton cycles have a very high efficiency, above 50%, even with dry-cooling [3], so their integration in a STPP can yield to an overall performance increase. Wang et al. [4] identified six possible supercritical cycles that can be integrated in a molten salt central receiver system with thermal storage: simple recovery cycle; recompression cycle; precompression cycle; intercooling cycle; partial-cooling cycle; and split expansion cycle. These cycles can be assessed according to different parameters, being the most important ones: the cycle efficiency; the complexity of the cycle compared to the most conventional one, the recompression cycle (represented in Figure 1); and the CO_2_ temperature increment in the source heat exchanger, as this value determines the molten salt volume in the solar field.

The intercooling cycle is the one with higher thermal efficiency when the thermal source temperature ranges from 600 °C to 800 °C, followed by the recompression cycle, that is also the simplest. Regarding the temperature difference in the source heat exchanger, the partial cooling layout is the one with the largest increment. In summary, three cycles can be identified to be the most suitable for coupling to a molten salt central receiver: recompression cycle, intercooling cycle and partial-cooling cycle [4,5]

The central receiver is usually a external-type with a surrounding heliostat field [1], although the cavity-type is recommended in recent investigations when the working temperature is high [2], because the radiation heat loss is lower compared to external receivers working at the same temperature. At last, the molten salt thermal storage consists of two tanks of molten salts, which have been sized to provide the nominal thermal power to the supercritical cycle for 6 h, with a charging time of 6 h.

This paper is focused in the heat exchanger between the solar field and the supercritical cycle, so a literature review on this heat exchanger is presented below.

There are several designs proposed in the literature for MS-to-CO_2_ heat exchangers, for both nuclear and solar applications, since both technologies use the scheme of a thermal source coupled to a supercritical CO_2_ cycle, as an alternative to increase performance.

The simplest design for this heat exchanger is a Shell and Tube Heat Exchanger (STHX), in which the CO_2_ at supercritical pressure circulates inside the tubes, and the molten salt trough the shell. This HX is well suited in supercritical cycles in which the source thermal energy is supplied through the low pressure side of the layout (85 bar approximately), as the one presented in [6]. Nevertheless, if a conventional supercritical cycle is used, the turbine inlet pressure should be limited to 200 bar, which constrains the cycle efficiency. There are several reasons that make the STHX not the most appropriate in conventional supercritical cycles: the great tube thickness due to the high pressure of the CO_2_ yields to a limited heat transfer and performance [7]; and, although the molten salt plugging does not occur in the shell, this fluid can be kept retained in the baffles and interstices of the HX, also yielding to a reduction in the heat transfer [8,9].

A more advanced design is the Printed Circuit Heat Exchanger (PCHE), which consists of plate sheets joined by diffusion-bonding, alternating hot-cold rows of semi-circular channels [10,11]. These microchannels withstand the high pressure of the CO_2_ (180–300 bar, approximately), and they also improve the heat transfer of this fluid, as the convection coefficient and the hydraulic diameter are inversely related. Nevertheless, PCHEs have the drawback of the viscous molten salt plugging in the microchannels. This issue has been studied in several reports of both nuclear [12] and solar [13] power plants, but very few designs address this problem. The most recent designs of MS-to-CO_2_ based on PCHE are focused on the heat transfer improvement by using airfoil fins in the microchannels [14,15,16].

Only one design has been found in the bibliography that tries to solve the problem of the molten salt plugging in microchannels [17]; the basic principle of this design is to face two plate sheets intended for molten salt, so that a circular channel is formed for this fluid, whereas the CO_2_ still circulates through semi-circular channels. Although this design does not optimize the heat transfer, the plugging and corrosion problems of the MS are reduced; nevertheless, the channel dimension for the molten salt is still small.

**Figure 2:**
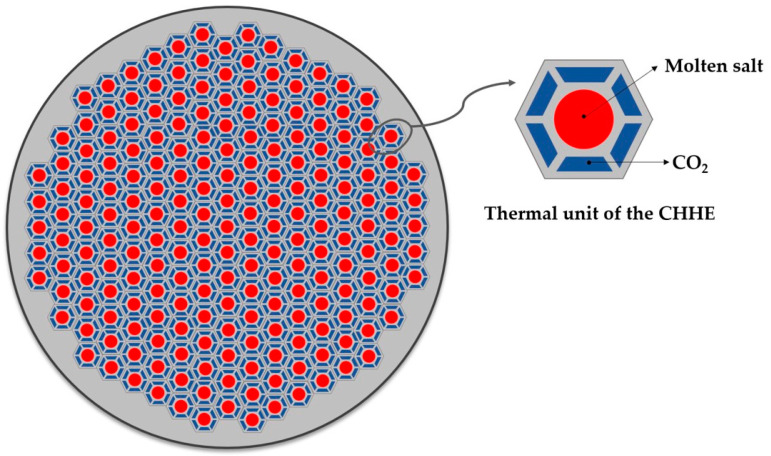
Cross section of the Compact Honeycomb Heat Exchanger and thermal unit.

To overcome the problems detailed in the two HX configurations described above, this work proposes and studies a new MS-to-CO_2_ HX design. From the analysis of the state of the art, it is clear that it would be desirable to increase the ratio of heat transfer area compared to the volume of the HX, that is, the most suitable design is a Compact Heat Exchanger (CHX); the lower convection coefficient of the supercritical phase is compensated by the larger area to transfer the thermal energy. But, at the same time, the MS channel must be larger enough to avoid plugging. To meet both conditions, a small compact shell and tube design [18] has been modified for the thermal duty and working pressure required by the supercritical cycle. The cross section of this design is a compact shell consisting of many thermal units like the one shown in Figure 2. The MS goes through a circular duct that is surrounded by 6 trapezoidal ducts, through which the CO_2_ circulates. Repetition of this unit gives the cross section of the shell a honeycomb-like appearance. Because of that, this HX will be referred as Compact Honeycomb Heat Exchanger (CHHE).

The thermo-mechanic model of this CHHE is explained in Section 2; an optimization of this design is accomplished in Section 3, by means of an exergy destruction minimization. As a result, the optimum working conditions of this design are set in Section 4.

## 2. Thermal Model and Boundary Conditions of the Compact Honeycomb Heat Exchanger

### 2.1. Thermal Inputs and Boundary Conditions from the Supercritical Cycle and the Solar Field

The CHHE is located between the supercritical cycle and the solar field as shown in Figure 1. The supercritical cycle is a conventional recompression cycle that is one of the three layouts (with the partial cooling and the intercooling) that show better characteristics to be coupled to a molten salt solar tower plant [4,5]. The cycle power output is 50 MW_e_, for which, the thermal energy in the CHHE is 100.99 MW_th_. Table 1 shows the thermodynamic properties of the state points following the numbering marked in Figure 1.

**Figure 3:**
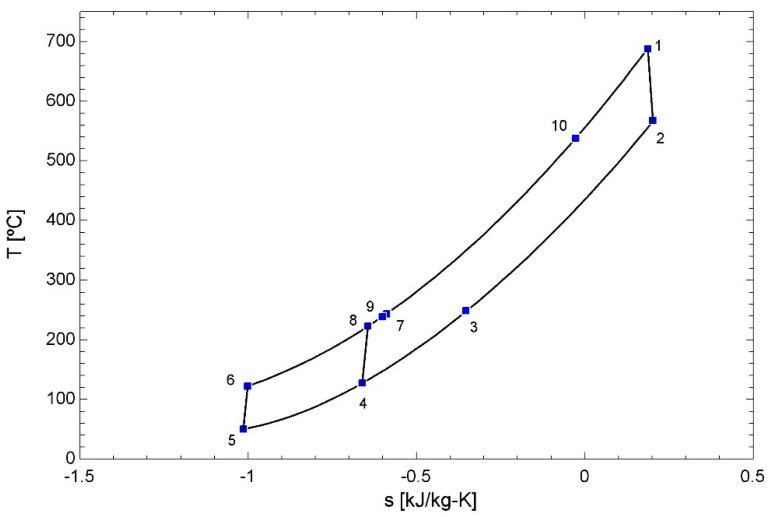
Temperature–entropy diagram for the sCO2 cycle, including the source heat exchanger.

Figure 3 shows the temperature-entropy diagram of this supercritical cycle. As it can be seen, this cycle is characterized by two compressors (processes 5, 6 and 4–8) and two recuperators, for low and high temperature (LTR and HTR, respectively). The source heat exchanger is characterized by process 10–1.

### 2.2. Heat Transfer Fluids and Mechanical Design of the CHHE

As the CHHE is located between the supercritical Brayton cycle and the solar field, the thermal fluids of this heat exchanger are the CO_2_ (cold side) and the molten salt (hot side). The CO_2_ thermodynamic properties have been obtained from NIST database [19]. On the other hand, the molten salt selected as HTF in the solar field and, thus, in the CHHE is a ternary chloride salt MgCl_2_/NaCl/KCl. This salt has a low melting point (385 °C) and a high thermal decomposition temperature (>800 °C), yielding to a large working temperature range; it has the cheapest estimated cost; and its volumetric heat capacity is higher, so its volume for a given thermal storage size is lower. Table 2 summarizes the main thermal properties of the ternary chloride molten salt selected [2].

The alloy selected for the CHHE is Haynes 242 (65%Ni-8%Cr-25%Mo, % weight). This alloy shows a good corrosion resistance in MS at temperatures higher than 700 °C, due to the higher percentage of molybdenum [20]. Besides that, the maximum allowable stress at the design temperature is very high, so it can withstand the high pressure difference at the working conditions [21]. The minimum thickness between the CO_2_ and the MS is calculated applying ASME codes [22].

The thermal power required in the source heat exchanger is 100.99 MW_th_, as pointed above. The CHHE has been divided in three modules of 33.66 MW_th_. The inlet temperatures of both the MS and the CO_2_ are also fixed by the solar field and the supercritical cycle, respectively. The CHHE is considered to be a balanced counter-flow heat exchanger.

There are two thermal inputs that will be parametrized for the optimization procedure in next section: the temperature approach (TA_MS-CO2_) between both streams, considering a balanced HX, and the pressure drop of the supercritical phase (dP_CO2_), which is the main pressure drop; once the CO_2_ pressure drop is fixed, the heat exchanger length is also fixed, and thus the MS pressure drop.

For a particular value of each of the previous two parameters (TA_MS-CO2_ and dP_CO2_), the outlet temperatures and the velocities of both streams are fixed. In this way, all the thermophysical properties that define the heat exchanger are established.

**Figure 4:**
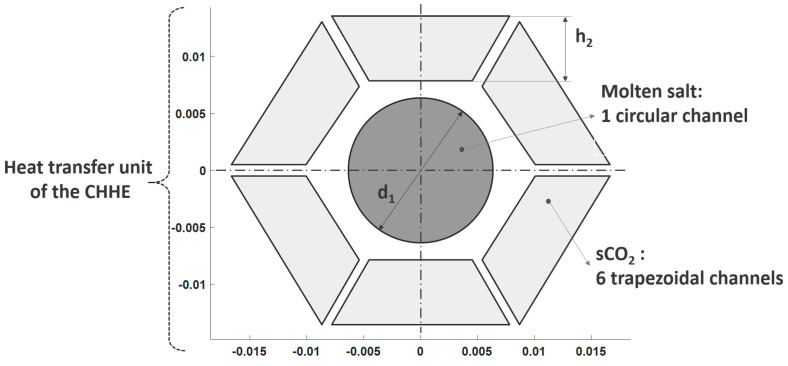
Dimensions of the thermal unit of the CHHE selected. (scale in mm).

Figure 4 represents a thermal unit of the CHHE accounting for the dimensions. Regarding the geometric parameters of the heat transfer unit, the MS channel diameter (d_1_ in Figure 4) has been set to 0.5 inch (12.7 mm), whereas the CO_2_ trapezoidal channel width (h_2_ in Figure 4) has been set to 5.7 mm. The trapezoidal shape of the duct cross section has been chosen because of geometric reasons, since in this way it is easy to form a thermal unit with a hexagonal shape whose repetition allows creating a honeycomb-like structure. The MS channel diameter is the minimum value reported in [23], that ensures the salt flow without clogging; and the CO_2_ channel dimensions have been selected to avoid a great pressure drop without penalizing the heat transfer due to a lower CO_2_ velocity. The pressure drop in the source heat exchanger of a STPP ranges from 2 to 3 bar, depending on the daily operation hours; this a greater value than that of baseload plants [24], that can operate continuously.

### 2.3. Thermo-Fluid Dynamic Model of the CHHE

Once geometric and thermal parameters are defined, the CHHE is calculated by a two dimensional thermo-fluid dynamic model. The heat exchanger is divided in N heat exchanger elements (HXEs), along the longitudinal direction, of the same thermal duty: Q˙HXE=Q˙N. In every element, the thermophysical properties of both fluids are assumed constant and equal to the average between the inlet and the outlet.

The overall heat transfer coefficient of the counterflow elementary CHHE, UHXE (Wm2·°C), is calculated by Equation (1).

(1) $$U_{HXE}=\frac{1}{\frac{1}{h_{conv1}}+\frac{1}{U_{w}}+\frac{1}{h_{conv2}}}$$

UW (Wm2·°C) is the thermal transfer coefficient for the wall between channels, that accounts for an equivalent constant thickness [25]; and *h_conv_* (W/m^2^/°C) is the convection heat transfer coefficient.

In this particular case, the flow, for both MS and CO_2_, is fully-developed turbulent (*Re* > 2300), so Gnielinski correlation is recommended [18], given by Equation (2).

(2) $$\begin{array}{c} \begin{array}{c} Nu_{Dh}=\frac{\left(f_{c}/8\right)\cdot \left(Re_{Dh}-1000\right)\cdot Pr}{1+12.7\cdot \left(\sqrt{\frac{f_{c}}{8}}\right)\cdot \left(Pr^{2/3}-1\right)}\cdot {\left(\frac{Pr}{Pr_{si}}\right)}^{0.11} \end{array}\\ \begin{array}{c} where: \end{array}\\ f_{c}={\left[1.82\cdot log\left(Re_{Dh}\right)-1.64\right]}^{-2} \end{array}$$

This correlation is valid for Reynolds numbers ranging from 2300 to 5 × 10^5^ and Prandtl numbers from 0.5 to 2000. In the above equation *f_c_* is the friction factor, calculated as needed from the Filonenko correlation [26]; *Re_Dh_* is the Reynolds number based on the inner hydraulic diameter; *Pr* is Prandtl number at the bulk fluid temperature; *Pr_si_* is the Prandtl number at the inner duct temperature, *t_si_*.

In case of laminar flow (only for molten salt under particular conditions), the Nusselt number is constant, as seen in Equation (3).

(3) $$Nu=4.3636\;for\;Re_{Dh}<2300$$

Once the value of the global heat transfer coefficient is known, length of every *HXE* is calculated by means of the basic equation of heat transfer, Equation (4).

(4) $${\dot{Q}}_{HXE}=U_{HXE}\cdot A_{HXE}\cdot \Delta T_{m}$$

${\dot{Q}}_{HXE}\left(W\right)$ is the thermal duty of every *HXE*; $A_{HXE}\;\left(m^{2}\right)$ is the heat transfer area of every *HXE*; $\Delta T_{m}\;$(°C) is the mean temperature, that is constant, as the CHHE is balanced and the CO_2_ is considered as an ideal gas. The length of every *HXE*, $L_{HXE}\;\left(m\right)$, is calculated from the heat transfer area, $A_{HXE}\;\left(m^{2}\right)$.

Finally, friction pressure destruction is calculated by means of the Darcy-Weisbach equation [18], for both fluids:

(5) $$\Delta P_{i}=\frac{1}{2}\cdot f_{D,i}\cdot \left(\frac{L_{HXE}}{D_{h,i}}\right)\cdot \rho_{ave,i}\cdot u_{ave,i}^{2}$$

where *D_h_ (m)* is the hydraulic diameter of the duct; *ρ* (kg/m^3^) is the average fluid density; *u* (m/s) is the average fluid velocity; and *f_D_* is the Darcy friction factor, that is calculated by the Techo et al. correlation [18], for turbulent flow ($10^{4}\leq Re_{Dh}\leq 10^{7}$); or the Hägen-Poiseulle correlation [18], in case of laminar flow ($Re_{Dh}\leq 2300$); finally, the subindex *i* refers to the each fluid: molten salt or CO_2_.

**Figure 5:**
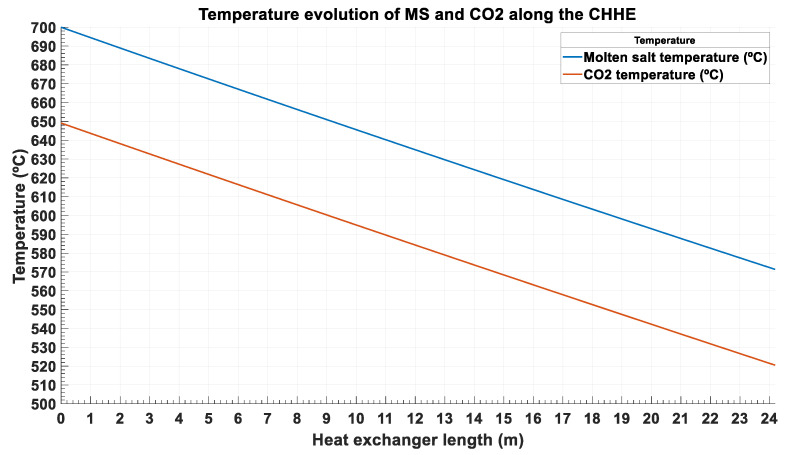
Temperature evolution of the ternary chloride molten salt and the CO_2_ along the CHHE.

Figure 5 shows the temperature evolution of the MS and the CO_2_ for a CHHE with a temperature approach T_AMS-CO2_ = 52 °C and a pressure drop dP_CO2_ = 2.75 bar. As it will be explained in next section, these values minimize the exergy destruction for the CHHE proposed.

**Figure 6:**
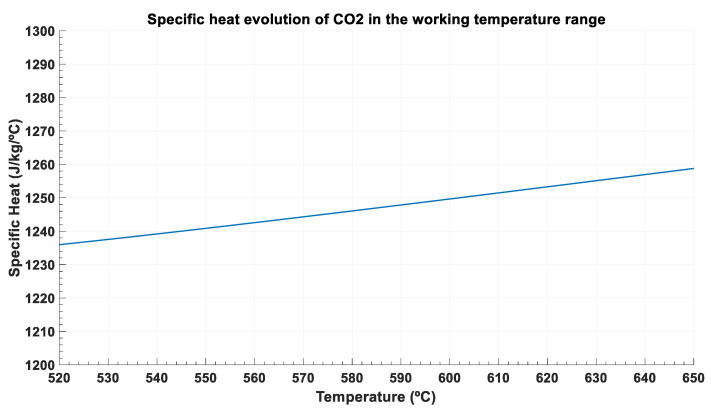
Specific heat evolution for the supercritical CO_2_ between 520 °C and 650 °C.

From Figure 5, it is concluded that temperature evolution is almost lineal for both streams, which means that specific heat is nearly constant. This is clear for the molten salt, which is an incompressible fluid approximately, but also for the supercritical CO_2_, as this fluid is at very high temperature and far away from the critical point. Figure 6 shows that specific heat ranges from 1.236 kJ/kg/°C to 1.258 kJ/kg/°C in the working temperature values (from 520 °C to 650 °C).

The main thermal and geometric parameters of this optimal CHHE are shown in Table 3. As seen in this table, this CHHE is divided into 6 smaller modules: 3 modules in parallel, in order not to have a shell diameter greater than 1 m; and 2 modules in series, for not exceeding 15 m long. As this HX is a new and not built design, it is difficult to set manufacturing restrictions, so these maximum values have been set according to the STHX limitation, in which the ratio shell diameter to length must be greater than 1/15.

The thermal model presented in this section has not been validated with empirical results, as it is a new design of heat exchanger. Nevertheless, the correlations used in this model, for both the CO_2_ and the ternary chloride molten salt have been validated in other HX designs for the same performance, by means of a numerical model in CFD, as the one presented in [17].

## 3. Optimization Procedure of the Heat Exchanger Based on the Exergy Destruction Minimization

The optimization of the CHHE proposed is based on the minimization of the exergy destruction, or entropy generation, in the heat exchanger. Assigning monetary values to these irreversibilities, this method allows to assess the cost of exergy destruction on each stream of the heat exchanger against its capital cost. Thus, the new objective function to be minimized is the Annual Total Cost (ATC), which takes into account the investment cost and the operation cost, including the irreversibilities in this last one.

(6) $$ATC=CRF\cdot CC+CELF\cdot C_{E}\cdot Y\cdot \Delta \dot{E}x_{destroyed}$$

In Equation (6), *CRF* is the capital-recovery factor and *CELF* is the constant-escalation levelization factor, both defined below; *C_E_* is the cost per unit of exergy ($/Wh), which has been taken as 0.00005$/Wh, according to several references [27,28]; *Y* is the yearly operation time, calculated for a solar multiple equal to 2: Y = 365*12 h; *CC* is the investment cost of the CHHE; finally, $\Delta \dot{E}x_{destroyed}$ is the total exergy destruction due to the most important irreversibilities in the heat exchanger.

The capital-recovery factor (CRF) and the constant-escalation levelization factor (CELF) are calculated by means of Equations (7) and (8).

(7) $$CRF=\frac{i_{eff}\cdot {\left(1+i_{eff}\right)}^{n}}{{\left(1+i_{eff}\right)}^{n}-1}$$

(8) $$\begin{array}{c} CELF=CRF\cdot \frac{k\cdot \left(1-k_{}^{n}\right)}{\left(1-k\right)}\\ \;\begin{array}{c} where \end{array}\\ k=\frac{1+r_{n}}{1+i_{eff}} \end{array}$$

In the above equations, *i_eff_* (%) is the weighted average capital cost, and *n* (years) is the economic life o span period of the power plant; r_n_ is the nominal escalation rate, which represents the annual change in cost and includes the effects of both the real escalation rate *r*_r_ and the inflation *r*_i_. The values of the parameters defined above are summarized in Table 4.

The next two subsections are devoted to the calculation of the Exergy Destruction ($\Delta \dot{E}x_{destroyed}$) and the Capital Cost (CC) of the Compact Honeycomb Heat Exchanger.

### 3.1. Accounting for the Exergy Destruction in the CHHE

Many researchers [29,30] have used the minimization of entropy generation, or exergy destruction, method to optimize the design of heat exchangers.

The causes of exergy destruction in a heat exchanger are: the finite temperature difference between hot and cold fluids, pressure drops on both fluids, and exergy losses associated to the non-adiabatic condition of a real heat exchanger, with a heat leakage with the environment; taking the limit of the system in the outer wall of the heat exchanger, these exergy losses constitute external irreversibilities to the heat exchanger.

In general, the total (internal and external) exergy destruction measured, normalized by the thermal power of the heat exchanger, is given by Equation (9).

(9) $$\frac{\Delta \dot{E}x_{d,total}}{{\dot{Q}}_{th,HX}}={\frac{\Delta \dot{E}x_{d}}{{\dot{Q}}_{th,HX}}]}_{\Delta T}+{\frac{\Delta \dot{E}x_{d}}{{\dot{Q}}_{th,HX}}]}_{\Delta P,h}+{\frac{\Delta \dot{E}x_{d}}{{\dot{Q}}_{th,HX}}]}_{\Delta P,c}+{\frac{\Delta \dot{E}x_{d}}{{\dot{Q}}_{th,HX}}]}_{loss}$$

These irreversibilities are calculated taking into account Gouy-Stodola Theorem [31], obtaining the equations summarized in Table 5, valid for ideal gas or incompressible liquid. For this exergy analysis, CO_2_ and MS can be assimilated to ideal gas and incompressible liquid, respectively. Dead state temperature (*T*_0_) has been taken as 298 K.

For the heat exchanger under study, the two main sources of exergy destruction are the finite temperature difference and the friction pressure drop on both sides, so these are the two irreversibilities that are going to be considered in the optimization procedure.

(10) $$\Delta \dot{E}x_{d,total}=\Delta \dot{E}x_{d}{}_{\Delta T}+\Delta \dot{E}x_{d}{}_{\Delta P}$$

### 3.2. Capital Cost Estimation of the CHHE

The capital cost of the CHHE is estimated by means of a base cost, *C_B_*, affected by three correction factors: pressure factor, *F_P_*, material factor, *F_M_*, and tube length correction factor, *F_L_* [32].

(11) $$CC_{HX}=F_{P}\cdot F_{M}\cdot F_{L}\cdot C_{B}$$

The base cost, *C_B_* ($), is calculated by Equation (12):

(12) $$C_{B}=exp\left\{11.0545-0.9228\cdot ln\left(A_{HX}\right)+0.09861\cdot {\left[ln\left(A_{HX}\right)\right]}^{2}\right\}$$

In Equation (12), $A_{HX}\;\left(ft^{2}\right)$ is the heat transfer area of the CHHE.

The pressure factor, F_P_, the material of construction factor, *F_M_*, and the tube length correction factor, *F_L_*, are given by Equations (13) and (14) and Table 6, respectively:

(13) $$F_{P}=0.9803+0.018\cdot \left(\frac{P}{100}\right)+0.017\cdot {\left(\frac{P}{100}\right)}^{2}$$

(14) $$F_{M}=a+{\left(\frac{A_{HX}}{100}\right)}^{b}$$

In Equation (13), P (psia) is the working pressure; in Equation (14), $A_{HX}\;\left(ft^{2}\right)$ is the heat transfer area of the CHHE, the constant *a* is equal to 9.6, and the constant *b* is equal to 0.06.

## 4. Results from the Optimization of the Compact Honeycomb Heat Exchanger

As said in Section 2, the optimization procedure to minimize the objective function Annual Total Cost (Mio.$) is done on the following parametrized thermal inputs: the temperature approach (TA_MS-CO2_) between both streams, and the pressure drop of the supercritical phase (dP_CO2_). TA_MS-CO2_ ranges from 30 °C to 60 °C, whereas dP_CO2_ ranges from 1.5 bar to 3.25 bar. These two parameters (pressure drop and temperature approach) have been considered in several optimization studies of heat exchangers, as they affect both the investment and the operation costs [33,34].

**Figure 7:**
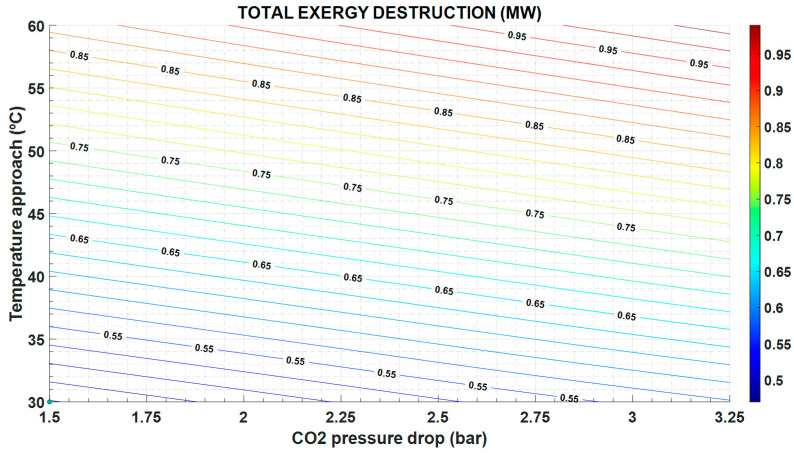
Exergy destruction in the CHHE, as function of the temperature approach between MS and CO_2_, and the CO_2_ pressure drop.

**Figure 8:**
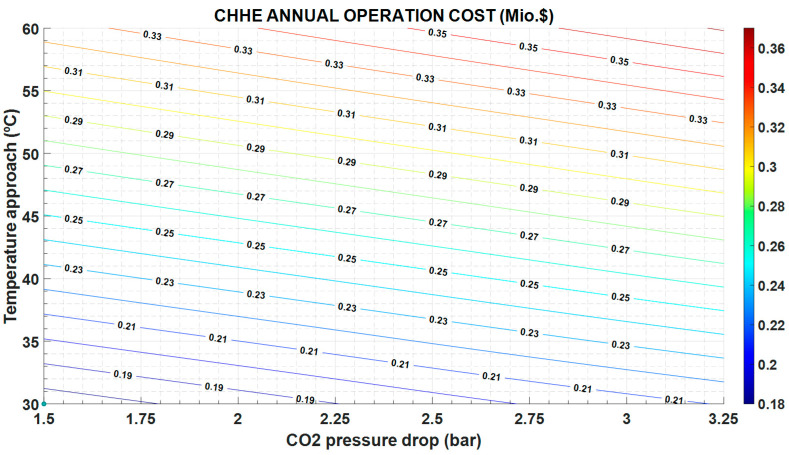
CHHE annual operation cost, as function of the temperature approach between MS and CO_2_, and the CO_2_ pressure drop.

**Figure 9:**
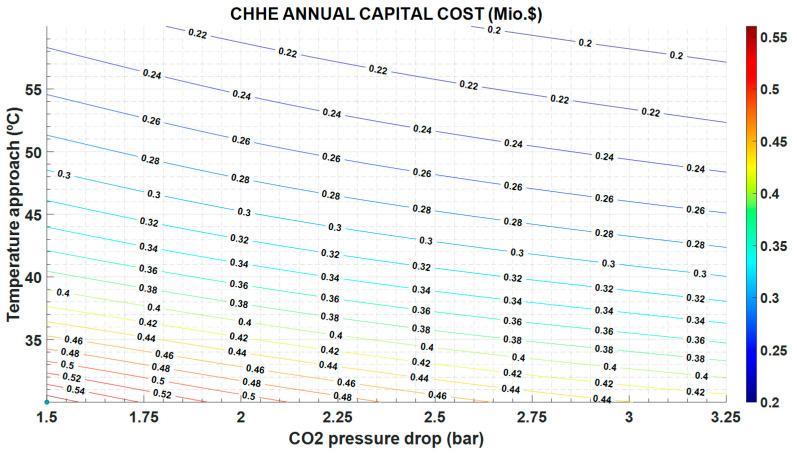
CHHE annual capital cost, as function of the temperature approach between MS and CO_2_, and the CO_2_ pressure drop.

The greater the temperature approach, the higher the exergy destruction (Figure 7) and the operation cost (Figure 8), but the lower the capital cost because the heat exchange area is also lower (Figure 9).

In the same way, the exergy destruction increases as the pressure drop increases, following expression in Table 5 and Figure 7. Instead, the greater the pressure drop, the lower the heat exchange area and the investment cost (Figure 9).

It is important to note that the exergy destruction increases with a greater pressure drop, since the entropy generation by friction also increases; and with the temperature difference between the two streams in the HX, as shown in the Figure 7.

The exergy destruction influences both the operation and maintenance cost and the investment cost. As can be seen in Figure 8, the operation and maintenance cost is proportional to the exergy destruction and follows the same variation: it increases with increasing exergy destruction. However, the investment cost in CHHE decreases as exergy destruction increases, as shown in Figure 9; in this case, a cheaper CHEE (due to smaller dimensions) also exhibits a more limited performance.

**Figure 10:**
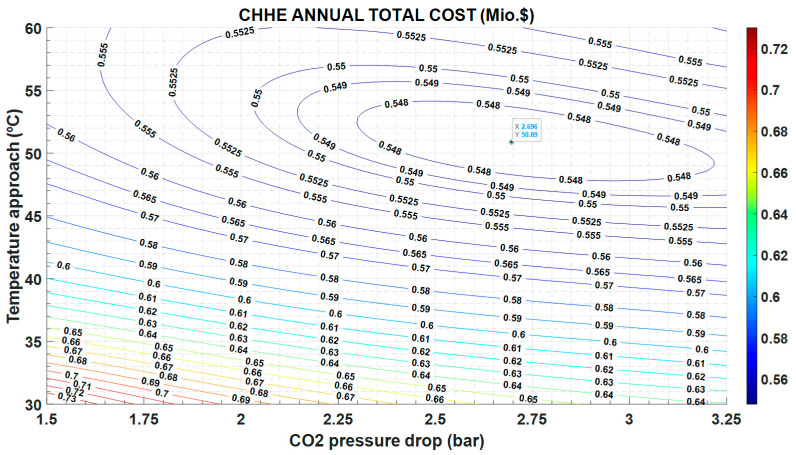
Annual Total Cost as function of the temperature approach between MS and CO_2_, and the CO_2_ pressure drop.

This different trend in O&M costs versus investment costs, yields to the minimum in the annual total cost observed in Figure 10.

Figure 10 plots the results of the optimization, showing that there is a minimum value for the ATC equal to 0.547 Mio.$, for 2.7 bar and 51 °C, approximately. These working conditions are very different of those reported in other type of HXs [24], in which pressure drop is 0.5 bar, whereas the temperature difference is 10 °C, approximately. It must be taken into account that this source heat exchanger only works 12 h a day, as it is located in a STPP, so cost derived of the irreversibilities in the operation are less penalized than if the power plant works continuously.

## 5. Conclusions

This work presents a new design for the source heat exchanger between the molten salt in the solar field and the supercritical CO_2_ in a Brayton cycle. This heat exchanger is compact, so the heat transfer of the supercritical phase in enhanced; and, at the same time, the molten salt duct is large enough to avoid plugging problems. This last characteristic is the main advantage of this design compared to other designs presented in the bibliography, since it allows the technological viability of this type of STPP based on a MS solar field and a sCO_2_ cycle.

The structure best suited to the above requirements is the honeycomb: the thermal unit of this compact heat exchanger consists of a circular channel for the molten salt, surrounded by 6 trapezoidal channels for the CO_2_. The thermal model of this new Compact Honeycomb Heat Exchanger (CHHE) is implemented and explained.

It is important note that the objective of this design is not to attain the highest heat transfer performance, but the technical feasibility of the heat transfer between a supercritical fluid, at high pressure, and a viscous liquid, that can cause plugging in the small channels of a CHX.

An economic optimization of the CHHE is also accomplished. Previous to this optimization procedure the main sources of exergy destruction have been identified: the temperature approach between both streams and the pressure drop on both fluids. If an exergy cost is defined and assigned to these irreversibilities, they can be included as an operation cost, that can be compared to the initial investment cost. In this way, the objective function Annual Total Cost is minimized as a function of the two thermal inputs: TA_MS-CO2_ and dP_CO2_. Results show that there is a minimum for 2.7 bar and 51 °C, approximately. These values are higher than others reported in bibliography, probably because the operational cost are lower, as the operation period of this STTP is lower than that of a conventional power plant.

Other future works include a numerical CFD model to validate the analytical model described in this paper, and to show possible thermo-mechanical problems. A later objective would be to build a laboratory scale model to obtain empirical results.

## Figures and Tables

**Table 1:** Thermodynamic properties of the state points of the recompression sCO_2_ cycle.

	Recompression Cycle		
	P (bar)	T (°C)	h (kJ/kg)
1	200	688	701.3
2	86.2	574.1	566.5
3	85.8	224.2	158.4
4	85.4	122.9	39.09
5	85	50	−80.9
6	201.2	118.3	−41.57
7	200.8	219.6	117.4
8	200.8	212	107.2
9	200.8	217.7	114.9
10	200.4	545.6	522.9
Cycle power (MW_e_)	50.00		
Source thermal power (MW_th_)	100.99		
Cycle efficiency (%)	49.57		

**Table 2:** Thermal properties of the ternary chloride salt MgCl_2_/NaCl/KCl. (Source: [2]).

Thermal Property	Correlation
Specific heat (J/kg/°C)	$c_{p}=1180$
Density (kg/m^3^)	ρ=1899.3−0.43·T(°C)
Thermal conductivity (W/m/°C)	k=0.5423−0.0002·T(°C)
Dynamic viscosity (Pa·s)	μ=8.25·10−6·exp(11874.71735(1350.84595+T(°C)))

**Table 3:** Main thermal and geometric characteristics of the optimal CHHE simulated.

CO_2_-MS COMPACT HONEYCOMB HEAT EXCHANGER	
**Sizing and geometric characteristics**	
Number of modules in parallel	3
Number of modules in series	2
Shell diameter of each module(m)	0.96
Length of each module(m)	12.09
Heat transfer area of each module (m^2^)	424.66
Number of channels of each module (MS)	440
Number of channels of each module (CO_2_)	2640
MS circular channel diameter (m)	0.0127
CO_2_ trapezoidal channel width (m)	0.0056
Material	Haynes-242
**Thermal characteristics**	
Thermal power (MW_th_)	33.664
Average global heat transfer coefficient (W/m^2^/°C)	1564.32
Temperature Approach (°C)	50.89
**Primary (Chloride molten salt)**	
Maximum velocity (m/s)	2.49
Primary inlet temperature (°C)	700
Primary inlet pressure (bar)	25
Primary mass flow rate (kg/s)	221.82
Primary outlet temperature (°C)	571.39
Primary outlet pressure (bar)	22.44
Primary pressure drop (bar)	2.56
Average convection heat transfer coefficient (W/m^2^/°C)	4360.55
**Secondary (CO_2_)**	
Maximum velocity (m/s)	10.27
Secondary inlet temperature (°C)	520.51
Secondary inlet pressure (bar)	202.68
Secondary mass flow rate (kg/s)	209.64
Secondary outlet temperature (°C)	649.11
Secondary outlet pressure (bar)	200
Secondary pressure drop (bar)	2.68
Average convection heat transfer coefficient (W/m^2^/°C)	2959

**Table 4:** Parameters for the thermo-economic analysis and optimization.

Economic Parameters	
Weighted average capital cost *i_eff_* (%)	7
Capital recovery factor CRF (%)	8.58
Nominal escalation rate (%)	5
k (%)	98.13
Constant escalation levelization factor (CELF)	19.74
Economic life (years)	25

**Table 5:** Equations to calculate normalized exergy destruction in a heat exchanger (Source: [32]).

Finite temperature difference	ΔE˙xdQ˙th,HX]ΔT=T0·(1Tc,lm−1Th,lm) where Tj,lm=Tj,i−Tj,oln(Tj,iTj,o), j=c or h
Pressure drops on hot and cold fluid sides	${\frac{\Delta \dot{E}x_{d}}{{\dot{Q}}_{th,HX}}]}_{\Delta P,j}=\frac{T_{0}}{T_{j,in}}\cdot \frac{1}{{\dot{Q}}_{th,HX}}\cdot {\left(\frac{\dot{m}\cdot \Delta P}{\rho_{in}}\right)}_{j},\;j=c\;or\;h\;$
Heat loss (to environment)	${\frac{\Delta \dot{E}x_{d}}{{\dot{Q}}_{th,HX}}]}_{loss}=\frac{{\dot{Q}}_{th,loss}}{{\dot{Q}}_{th,HX}}\cdot \left(1-\frac{T_{0}}{T_{h}}\right)$

**Table 6:** Parameters for the thermo-economic analysis and optimization.

Tube Length (ft)	F_L_
8	1.25
12	1.12
16	1.05
20	1.00

## Nomenclature

**Acronyms**	
*ATC*	Annual Total Cost
*CELF*	Constant-escalation levelization factor
CFD	Computational Fluid Dynamics
CHHE	Compact Honeycomb Heat Exchanger
CHX	Compact Heat Exchanger
*CRF*	Capital-recovery factor
DOE	U.S Department of Energy
HX	Heat Exchanger
MS	Molten salt
PCHE	Printed Circuit Heat Exchanger
SHX	Source Heat Exchanger
STHX	Shell and Tube Heat Exchanger
STPP	Solar Thermal Power Plant
TES	Thermal Energy Storage
**Latin letters**	
*A*	Area (m^2^)
c	Specific heat (J/kg/°C)
*C*	Cost ($)
*CC*	Capital Cost ($)
*C_E_*	Cost per unit of exergy ($/Wh)
*C_B_*	Base cost ($)
*D_h_*	Hydraulic diameter (m)
*d*	Diameter (m)
dP	Pressure Drop (Pa)
$\dot{E}x$	Exergy (W)
*f*	Darcy pressure friction loss factor
*F*_P_	Pressure factor
*F_M_*	Material of construction factor
*F_L_*	Tube length correction factor,
*h_conv_*	Convection heat transfer coefficient (W/m^2^/°C)
*i_eff_*	Weighted average capital cost
*k*	Thermal conductivity (W/m/°C)
*L*	Length (m)
$\dot{m}$	Mass flow rate (kg/s)
*N*	Number of heat exchanger elements
*n*	Number of years
*Nu*	Nusselt number
*p*	Pressure (Pa)
*Pr*	Prandtl number
$\dot{Q}$	Thermal power (W)
*r_n_*	Nominal escalation rate
*Re*	Reynolds number
*T*	Temperature (°C)
*TA*	Temperature Approach (°C)
*U*	Overall heat transfer coefficient (W/m^2^/°C)
*u*	Velocity (m/s)
*V*	Volume (m^3^)
*Y*	Yearly operation time
**Greek Letters**	
ρ	Density (kg/m^3^)
μ	Dynamic viscosity (Pa·s)
**Subscripts**	
*ave*	Average
*net*	Net
*p*	Pressure
*th*	Thermal
